# Finite Element Analysis for Surface Acoustic Wave Device Characteristic Properties and Sensitivity

**DOI:** 10.3390/s19081749

**Published:** 2019-04-12

**Authors:** Tao Wang, Ryan Green, Rasim Guldiken, Jing Wang, Subhra Mohapatra, Shyam S. Mohapatra

**Affiliations:** 1 James A Haley VA Hospital, Tampa, FL 33612, USA; taowang@mail.usf.edu; 2 Center for Research and Education in Nanobioengineering, University of South Florida, Tampa, FL 33612, USA; rgreen1@health.usf.edu (R.G.); guldiken@usf.edu (R.G.); 3 Department of Internal Medicine and Pharmacy Graduate Programs, University of South Florida, Tampa, FL 33612, USA; 4 Department of Molecular Medicine, University of South Florida, Tampa, FL 33612, USA; 5 Microfluidics and Acoustics Laboratory, Department of Mechanical Engineering, College of Engineering, University of South Florida, Tampa, FL 33610, USA; 6 Department of Electrical Engineering, University of South Florida, Tampa, FL 33610, USA; jingw@usf.edu

**Keywords:** surface acoustic wave (SAW), finite element method (FEM), sensitivity, IrO_2_, ZnO

## Abstract

The most vital step in the development of novel and existing surface acoustic wave (SAW)-based sensors and transducers is their design and optimization. Demand for SAW devices has been steadily increasing due to their low cost, portability, and versatility in electronics, telecommunications, and biosensor applications. However, a full characterization of surface acoustic wave biosensors in a three-dimensional (3D) finite element model has not yet been developed. In this study, a novel approach is developed for analyzing shear horizontal Love wave resonator devices. The developed modeling methodology was verified using fabricated devices. A thorough analysis of the 3D model and the experimental device was performed in this study including scattering parameters (S-parameters), reflection coefficient parameters, transmission parameters, and phase velocity. The simulated results will be used as a design guideline for future device design and optimization, which has thus far resulted in close matching between prediction and experimental results. This manuscript is the first to demonstrate a 3D finite element model to correlate the sensitivity of the SAW device with the magnitude of the phase shift, the real and imaginary part of the response, insertion loss, and the frequency shift. The results show that the imaginary part of the response shift has a higher sensitivity compared to other parameters.

## 1. Introduction

Surface acoustic wave (SAW)-based devices have been mass-produced and widely used in consumer products and communication equipment [1]. The increasing demand for fast and precise simulation tools is driven by their essential role in research and development. The advantages of these tools include reduced time and cost for production as well as improved design optimization and a better understanding of the underlying Multiphysics [2]. Due to their high sensitivity and great portability [3], SAW devices can be utilized in a wide range of applications including electronic actuators [4,5], telecommunication modulators [6,7], as well as biochemical and gas sensors [8,9,10,11,12]. A number of simulation techniques and methods have been developed and applied to acoustic modeling but there is still need for significant improvement [2]. The most effective methods are based on numerical analysis such as Green’s function and the coupling-of-modes method, which are widely used to analyze and optimize SAW device designs [13,14,15]. Other methods for analyzing SAW devices are based on impulse response, transmission matrices, and equivalent circuit models, but they are not able to accurately reproduce all the behaviors of SAW devices—especially estimating all the electromechanical effects and multiple order effects on the device’s function [16,17,18,19]. Many years ago, a research team proposed the use of cascaded equivalent circuits for the SAW interdigital transducer (IDT) [20]. Over the last few years, research has been conducted on mass loading (the energy storing effect), and this model has been widely used in SAW research and development due to its simplicity and reasonable accuracy [21]. To save computation resources and time, several techniques have been developed to characterize SAW properties for the thin metal layer and metallic grating structures [22]. Effects of the grating finger thickness have also been studied extensively [23]. Even though the Green’s function and coupling-of-modes methods can be used to estimate the multiple order effects, it becomes more complicated for them to stimulate and estimate the effects of multiple guiding layers, especially with multiple piezoelectric layers. However, because of limited computer resources, early research in this field and the modeling tools it employed were relatively simple such as the perturbation theory model [24]. The recent rapid progress of computer hardware and software technologies has made it possible to deal with very complex problems with multiphysics coupling and multilayer metallic grating structures [5,25]. However, these methods have significant limitations and require multiple assumptions that limit their applicability while solving physical constraints, boundary conditions, complex geometry materials, and multiphysics coupling.

Previous research [26,27,28] has led to an effective finite element method (FEM) analysis technique which utilizes an impulse signal through an applied voltage to propagate at the surface of the device and transfer the energy from input to output interdigital transducers to analyze the frequency response. The results obtained from this technique can help the researcher predict the device’s capability/sensitivity more accurately and to optimize the device coupling with different guide layers and samples. Fluids with particles of different concentrations and viscosities resulting in frequency shifts in SAW devices are also being investigated using the commercial simulation package (COMSOL Multiphysics) [29] with the FEM. This analysis can easily predict experimental results and optimize the design in an affordable way. However, most of the reports are focused on the wave reflection–transmission and mechanical deformation [30,31]. The mass sensitivity and other electromechanical properties have not been demonstrated based on FEM, and its experimental verification has also not been adequately reported until now.

In this study, multiple electromechanical properties and mass sensitivity effects determined by the guide layer thickness are discussed. A 3-dimensional (3D) model based on a realistic device is built to obtain 3D wave responses. Due to the limitations of the computational resources available, this model was designed with a trade-off between computation speed and precision. By comparing the calculated results from the simulation to measured results from network analysis, we show that the simulation results can be in very close agreement with the measured responses in multiple electrical properties such as S-parameters, reflection parameters, transmission parameters, and velocity phase. The calculated phase sensitivity and frequency shift sensitivity are compared in order to optimize signal measurement methods. The most three common electronic configurations to measure the mass loading are based on the oscillator circuit, vector voltmeter, and network analyzer [32]. Oscillatory circuit systems can be designed to measure the frequency shift or phase shift in a loop system. The vector voltmeter can be used to measure the phase shift. The network analyzer is the instrument used to characterize the device for all information such as phase, frequency, imaginary response, real response, and standing wave ratio. The optimized measurement method from the simulation can help the researcher determine the optimal measurement configuration for use in the physical device. The sensitivity of the same device at different frequencies will be differently evidenced by its S-parameters. The novel approach proposed herein demonstrates a new path for optimizing SAW sensor models with more accurate parameter selection, which can be used to predict the sensitivity and response of different guiding and mass loading layers for multilayer device optimization. The thorough comparison between phase shift sensitivity, insertion loss changes, and frequency shift sensitivity, shows that phase shift is a more sensitive parameter than the rest.

## 2. Experiment and Methods

### 2.1. Background

Recently, an increasing number of research groups have shown interest in finite element analysis of SAW devices with varying structures and designs. In this study, all simulation models were based on 36° YX cut lithium tantalate substrates, employed the commercially available finite element analysis package (COMSOL Multiphysics 5.2), and were analyzed in Matlab^©^. Two computers with E5-2630 V3 processors and 96 GB RAM were used to calculate the results, and the calculation time for the model was greater than 72 h. The method employed not only provides information on filter input–output signal levels, phase velocity, wave phase, wave interference, and diffraction, it also renders excellent preliminary design information on the response of a SAW sensor [33]. The frequency analysis of a SAW device was conducted through a full three-dimensional (3D) model simulation. The 36°YX cut lithium tantalate functions to generate the shear horizontal surface acoustic wave. As the guiding layer (such as SiO_2_ or ZnO) was added to the top surface, the Love wave was generated and propagated at the guiding layer. A fundamental simulation demonstrated the signal response in the form of scattering parameters (S-parameters), reflection parameters, transmission parameters and phase.

### 2.2. Model Structures

The relationship between piezoelectric stress, strain, electric field, and electric displacement field was deduced by stress–strain relationship equations in non-piezoelectric materials after the voltage was applied. The electric field *E* will cause a change of piezoelectric materials’ molecular charge distributions, which will result in a surface charge buildup. In the current study, the following equations were used in COMSOL to model surface charge density and mechanical stress relationship in stress-charge form as shown below [34]:

(1) D=[e][S]+[ε]E

(2) $$\left[T\right]=\left[c\right]\left[S\right]-\left[e^{t}\right]E$$

In the Equations (1) and (2), [*e*] represents the piezoelectric constant matrix, *E* is the applied electric field, [*c*] is the elastic constants, [*ε*] is the dielectric permittivity, and [*S*] is the strain matrix. The $\left[e^{t}\right]$ matrix is the 3 × 6 transposed matrix of the piezoelectric constant matrix [34].

**Figure 1:**
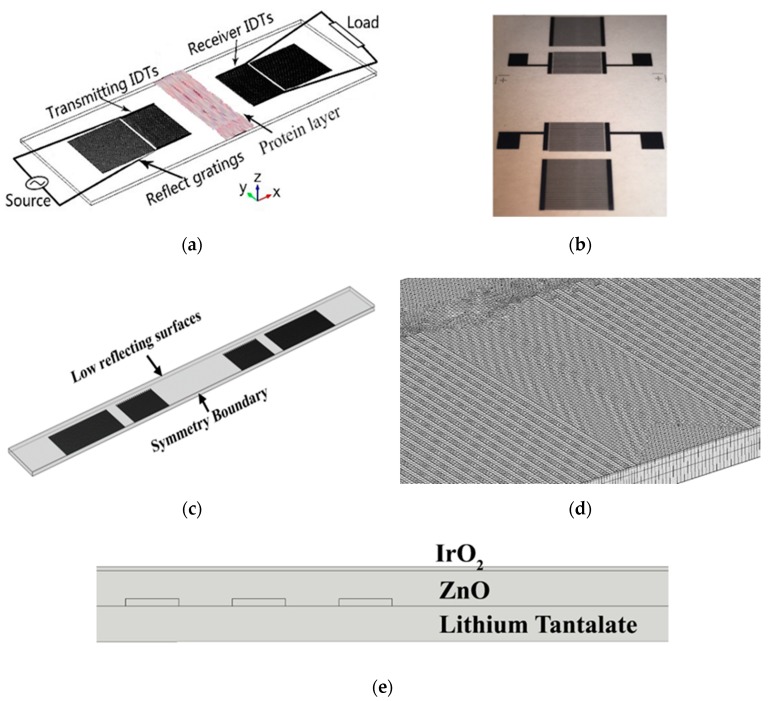
(**a**) Schematic diagram showing the design and structure of the real SAW device. (**b**) Fabricated device with only the finger layer. (**c**) The 3D model of the device used in COMSOL. (**d**) The completed mesh of the model. (**e**) Conceptual view of the double guide layer of the SAW device.

The 3D modeling structure in Figure 1c was selected in this study. The simulation model in Figure 1c has 20 pairs of interdigital transducers (IDTs) and 30 pairs of reflecting fingers for both receiving and transmitting ports. The realistic device and simulation model have the same wavelength (*λ*) of 298 μm and same delay line length of 38.25*λ*. The reflecting fingers were located 10.25*λ* away from the IDTs. Substrate thickness was 500 μm. IDT parameters used in the design are listed in Table 1. The first layer of the device was the chrome IDT fingers with a thickness of 100 nm, the second layer was the waveguide layer made of 500 nm-thick ZnO, and the top layer was the IrO_2_ layer as seen in the fabricated device in Figure 1c. The layer’s properties and fabrication process are shown in our previous report [35].

### 2.3. Boundary Conditions and Meshing

A time-domain analysis was conducted to calculate the dynamic characteristics of the device in response to a short impulse signal. The impulse voltage was applied to the input electrodes, where *V*_+_ and *V*_−_ were applied to the even and odd fingers in Equations (3) and (4). For this simulation, a step size of 2 ns from Equation (5) and a total simulation period of 8800 ns were used. The material properties used in the simulation are shown in Table 2.

(3) $$V_{+}=\{\begin{array}{c} +0.5\;V,\;0\leq t\leq 2\;ns\\ 0\;V,\;t>2\;ns \end{array}$$

(4) $$V_{-}=\{\begin{array}{c} -0.5\;V,\;0\leq t\leq 2\;ns\\ 0\;V,\;t>2\;ns \end{array}$$

(5) $$T_{v}<\frac{1}{20\cdot f_{max}}$$

The boundary condition of the output electrodes was set to an initial voltage of zero to an outside terminal, which is also connected to the voltmeter and load. The bottom surface and side surface of the substrate were both set to be low reflecting surfaces as the absorber. A symmetry boundary was used in the model as illustrated in Figure 1c. Initial body displacement fields in X, Y, and Z directions (Ux, Uy, and Uz) were set to zero. The measured voltages generated on the output electrodes were used to calculate the frequency response of the device. The output electrode finger was connected to an electrical circuit via the terminal boundary. A quadrilateral mesh was applied on the fingers, and the triangular mesh was used on the rest of the top surface with swept mesh to the whole device. The device was meshed with different node densities to verify the frequency independence from the mesh size.

**Figure 2:**
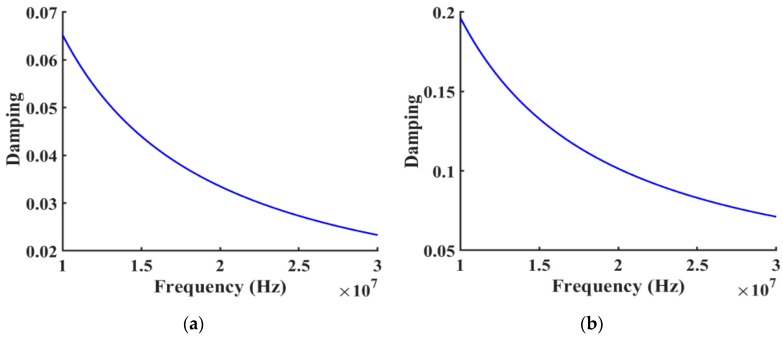
(**a**) The applied mechanical damping ratio in the substrate layer (lithium tantalate) at different frequencies. (**b**) The applied mechanical damping ratio in the IrO_2_ layer at different frequencies.

The damping effect caused by mass loading is a challenge for accurate model generation because the damping ratio is not only mass dependent, but also a frequency dependent parameter that cannot be readily implemented across an extended range of the frequencies. As the mass of the IrO_2_ layer increased, the insertion loss (S21) and the difference between simulation and physical measurements also increased due to a mechanical loss factor being applied to the model in the form of the loss factor (also known as damping ratio) on the IrO_2_ and lithium tantalate layers in Figure 2. This loss factor was calculated from the experimental results using the equation η=1/2Q where *Q* is the quality factor of the device. The performance assessment of most acoustic wave sensors was essentially determined by quality-factor and device sensitivity. The quality factor (*Q*) is a measure of how capable the acoustic wave device is of retaining its energy during oscillation. *Q* factor is defined as the ratio of the energy stored in the resonator to the energy dissipated per cycle. The energy losses will be the major parameter affecting the *Q* factor, which could include anything from a mechanical damping loss to thermal elastic loss, while also depending on the loss in the electrical domain, such as coupling loss, dielectric loss, and conductive loss which is negligibly small in this case.

### 2.4. Frequency Response Calculation

In this study, a discrete fast Fourier transform (FFT) function was performed after the waveform of the output voltage and charge were imported into Matlab^©^. At the same time, the logarithmic frequency response was converted to the admittance (z) of the device in the frequency domain [27]. After admittance was calculated, a full analysis of the model was performed including the wave phase, linear magnitude, standing wave ratio (SWR), the imaginary part of the response, and real part of the response, which were determined by the equations in Table 3.

### 2.5. Design and Fabrication

Devices with different guide layers were fabricated and tested to compare their frequency responses to the simulation results. All devices were fabricated using conventional MEMS fabrication processes. The ZnO layer was sputtered by a radio-frequency (RF) sputtering system (AJA) with a 99.9% ZnO target. The oxygen and argon flow ratio were kept at 1:1 and the wafer temperature was set to 180 °C. IrO_2_ was also sputtered by an RF sputtering system (CRC) with a 99.99% IrO_2_ target at room temperature in a pure argon environment. The frequency responses of the devices were measured using a vector network analyzer (Anritsu 37369A) of 50 Ω characteristic impedance.

## 3. Results and Discussion

### 3.1. FEM Analysis of a Multi-Layer SAW Device

**Figure 3:**
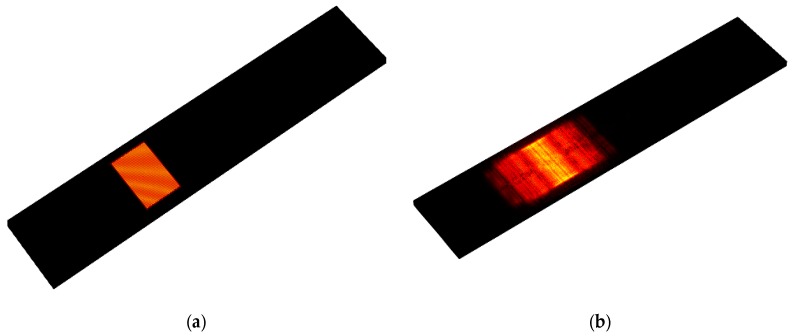
(**a**) Time-lapse elastic displacement distribution in the SAW device after an initial pulse voltage was applied to the input IDTs at T = 0 ns. (**b**) Time-lapse elastic displacement distribution at T = 300 ns.

**Figure 4:**
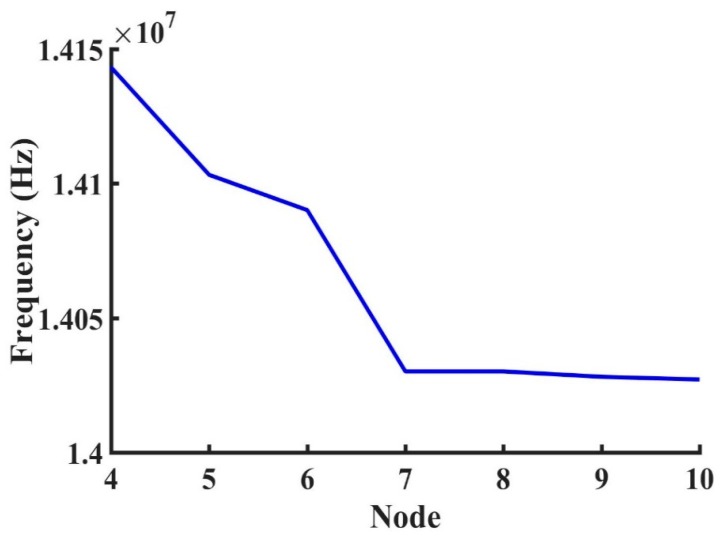
Effect of node density (mesh node in a wavelength).

**Figure 5:**
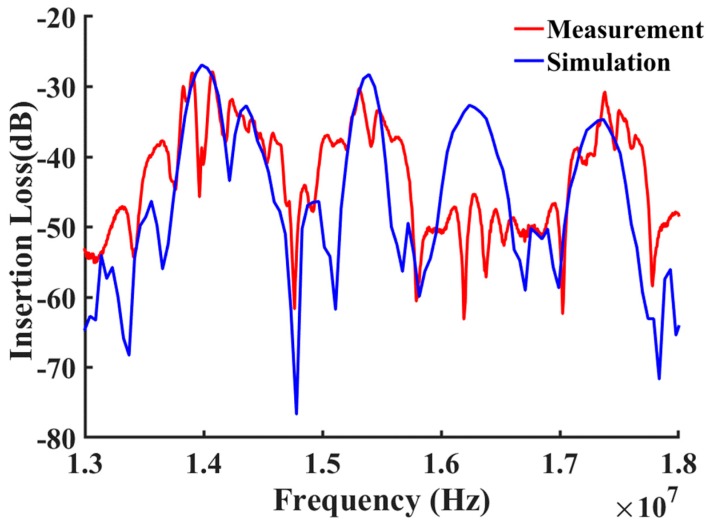
Comparison between simulated and measured frequency responses for the device (with 100 nm Cr IDTs only) from 12 MHz to 18 MHz.

**Figure 6:**
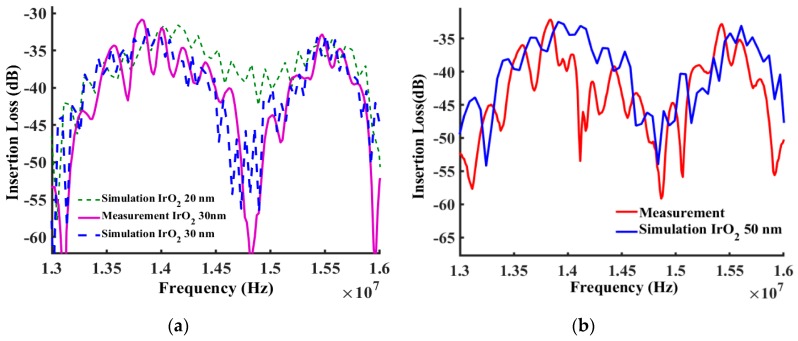
(**a**) Comparison between simulated and measured frequency responses for the device (with 100 nm Cr IDTs, a 500 nm-thick ZnO layer, and an IrO_2_ layer of 30 nm) from 13 MHz to 16 MHz. The experimental results are obtained from the device with Cr IDTs, a 500 nm-thick ZnO layer, and a 30 nm-thick IrO_2_ layers. (**b**) Comparison between simulated and measured frequency responses for the device (with 100 nm Cr IDTs, a 500 nm-thick ZnO layer and a 50 nm-thick IrO_2_ layer) from 13 MHz to 16 MHz.

**Figure 7:**
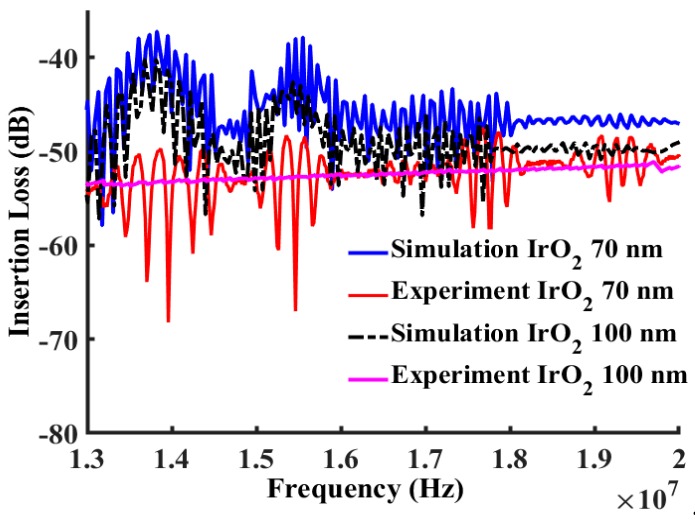
Comparison between simulated and measured frequency responses for the device with Cr IDTs, a 500 nm-thick ZnO layer, and a 70 nm/100 nm IrO_2_ layer from 13 MHz to 20 MHz.

The propagated shear horizontal waves traveled through the path line as shown in Figure 3. As shown by the modeled elastic displacement in Figure 3, the waves traveled through the active device region and were reflected by the edges. A mesh independent study in Figure 4 shows that the frequency response was independent of mesh size when more than six mesh nodes were created per wavelength. The device was meshed with a node density of seven nodes per wavelength, and more than 10 × 10^6^ degrees of freedom in total were maintained sweeping throughout all the different studies in this manuscript (Average mesh size quality of 0.968). The frequency spectrum was converted from the wave received by the output electrodes and then plotted out to compare with the measurements as shown in Figure 5, Figure 6 and Figure 7. A guiding layer was applied to the acoustic wave devices to improve the sensitivity, temperature stability, and electromechanical coupling coefficient [37]. As the wave propagates, its phase velocity depends on the properties of the different guide layers, the effect of the multi-guide layer material is investigated in Figure 5. A piezoelectric layer with a relatively high permittivity added on top of the piezoelectric layer (Lithium Titanate) increases the electromechanical coupling, thus allowing fabrication of devices with reduced insertion loss [37]. After the ZnO waveguide layer was deposited on top of the surface of the Lithium Tantalite substrate, a layer of IrO_2_ was deposited on top of the ZnO layer to further increase the sensitivity [35]. The dielectric properties of different guide layers will also affect the wave properties in a different fashion. The comparison between the simulation results and experimental measurements illustrated in Figure 5, Figure 6 and Figure 7 shows that the predictions of the 3D model are reasonably comparable to the experimental frequency spectrum of the devices.

In Figure 5, the simulation results show a very similar resonance frequency (14.03 MHz) compared to the measured results (14.05 MHz). The trend of the simulation results at the first two modes with peak frequency at around 14 MHz and 15.3 MHz, were very close to the experimental results, but the third peak frequency did not match the measurement as well as the first two modes. The insertion loss of the simulation results was smaller than the experimental measurement. The major reason for the third peak mismatch in Figure 5 is the different reflecting behavior of the waves reflected by the edge of the model compared to the edge of the real device. The real devices had a long distance between the side edges and the IDTs (~40*λ*). To reduce the required computation time and resources, this distance was changed in the simulated device to 4*λ*. After reducing the distance between the IDTs to the edge, and applying the absorption boundary, the reflection behavior and wave interference were affected. The reflection behavior difference contributes to this variation between simulation and experiment on the third peak, which may also be interfered by other waves on the physical device that are not present in the simulation study. The measured resonance peak at 14.05 MHz with a 28.7 dB of insertion loss showed a larger diversion from the simulation results of an insertion loss of 26.9 dB at 13.98 MHz.

As shown in Figure 6a, the simulated frequency characteristic did show a good match with the experimental data in the previous research [35]. In Figure 6 and Figure 7, we show that as the IrO_2_ layer increased, the insertion loss decreased. The simulation results measuring insertion loss showed a very good match with the measurement for the device with 100 nm Cr IDTs, a 500 nm-thick ZnO, and a 30 nm-thick IrO_2_ layer. Figure 7 shows that as the layer of the IrO_2_ increased, the insertion loss increased since the waves began to attenuate or cease propagation. In the experimental measurements, as the layer of the IrO_2_ increased to 100 nm on top of the 500 nm ZnO layer, the wave propagation vanished. At 100 nm thickness all frequency response was eliminated because of IrO_2_’s electrical properties. The thin layer of the IrO_2_ had a large resistance, which can be considered as a non-conductive layer. As the IrO_2_ thickness increased, the conductive IrO_2_ shorted the device, which can cease the acoustic wave. In this simulation, IrO_2_ was considered as a nonconductive elastic material to simplify the problem and characterize mass sensitivity. The abrupt transition of IrO_2_ from high resistance to conductive, which was observed in the experimental results, was not incorporated into the simulation.

### 3.2. Conversion of Complex into Real Quantities

After the simulation data were generated, a custom written Matlab^©^ program read the file and converted the data to be used in different Cartesian diagrams.

**Figure 8:**
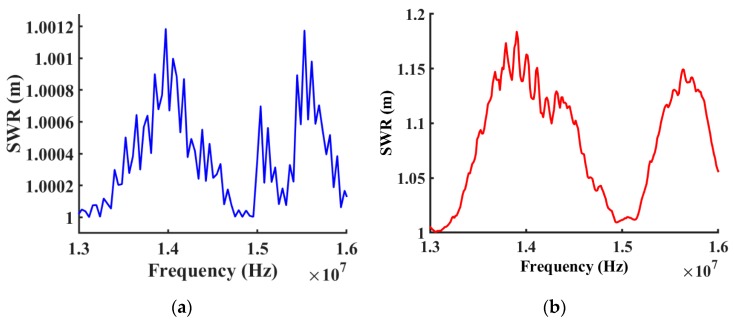
Comparison between simulated (Blue) and measured (Red) standing wave ratios of frequency response for the device with Cr IDTs only, without coatings, from 13 MHz to 16 MHz. (**a**) Simulated standing wave ratios of the design. (**b**) Measured standing wave ratios from the fabricated device.

The standing-wave ratio (SWR) represents a mathematical expression of the non-uniformity of an electrical field throughout a transmission line at radio frequencies. SWR is defined as the ratio of maximum radio-frequency (RF) voltage to minimum RF voltage along the line [38], which is also known as the voltage standing-wave ratio (VSWR). The voltage on a signal transmission line is the same at all points on the line when power losses caused by line resistance and imperfections in the dielectric material separating the line conductors are assumed to be negligible. SWR of the surface acoustic device is mathematically related to the input power and reflected power when the device is tested via a network analyzer. In an ideal scenario, SWR is 1:1 when there is no power loss or reflected power. Figure 8 shows a reasonable comparison between simulated SWR and experimentally measured results over a range of frequencies.

**Figure 9:**
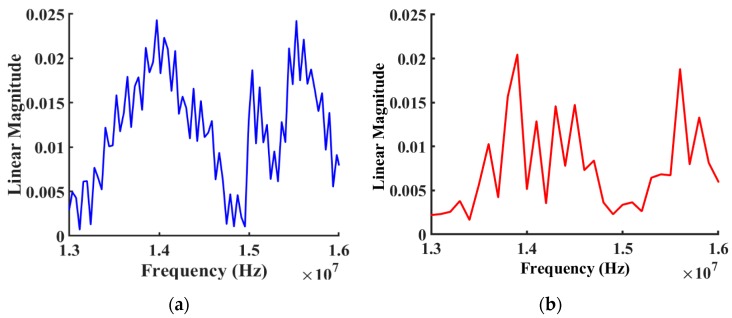
Comparison between simulated (Blue) and measured (Red) linear magnitude frequency response for the device (with Cr IDTs only) from 13 MHz to 16 MHz. (**a**) Simulated linear magnitude frequency response of the design. (**b**) Measured linear magnitude frequency response from the fabricated device.

Figure 9 presents the linear magnitude of forwarding transmission vs. frequency (S21). After the data were transferred to the Laplace and Fourier domains, the magnitude and phase responses were obtained—which are commonly referred to as the frequency response. The first two wave modes matched the experimental measurements very well, where the linear magnitude of 0.0248 at the first simulated peak was fairly close 0.0232 from the corresponding measurements. However, the simulation illustrated additional spurious modes that were not present in the experimental measurements.

**Figure 10:**
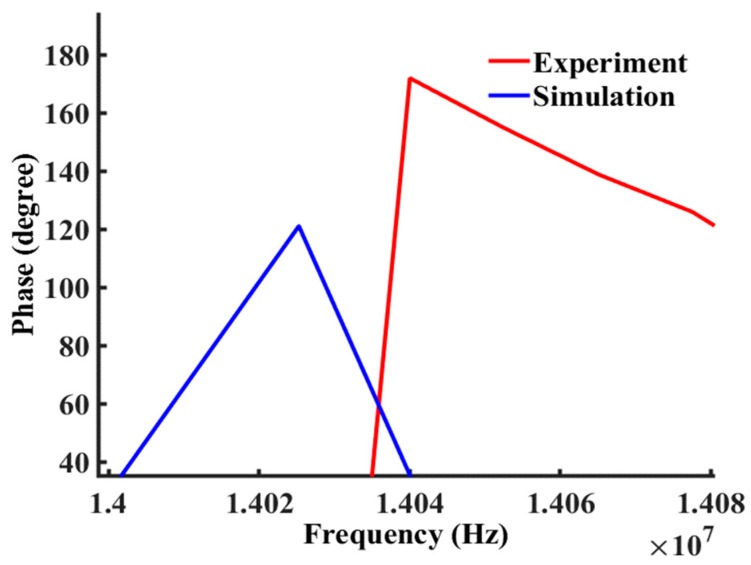
Comparison between simulated (Blue) and measured (Red) phase angle responses from the device (with Cr IDTs only).

The insertion phase angle versus frequency is another very important characteristic of the device, which is also critically important for analysis and detection. Most prior work only reported results based on phase angle shift to different mass loading [39,40]. After the complex number of the propagated wave is calculated from the voltage and current of the output electrodes, the phase angle of z can be calculated from the equation listed in Table 3. The simulated phase angle is compared with the experimental measurements to verify the model in Figure 10.

**Figure 11:**
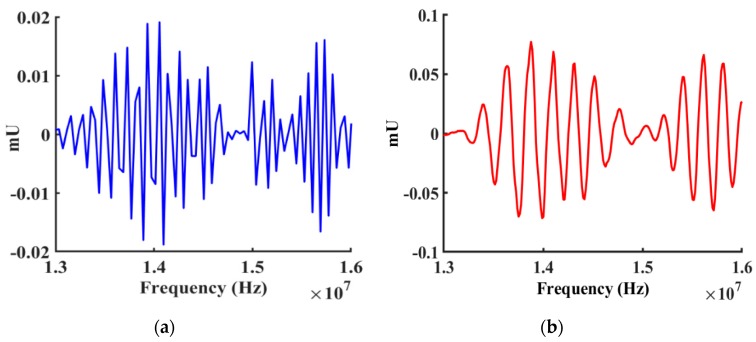
Comparison between simulated (Blue) and measured (Red) imaginary magnitudes for the frequency response of the device (with Cr IDTs only) from 12 MHz to 16 MHz. (**a**) Simulated imaginary magnitude of the response from the design. (**b**) Measured imaginary magnitude of the response from the fabricated device.

**Figure 12:**
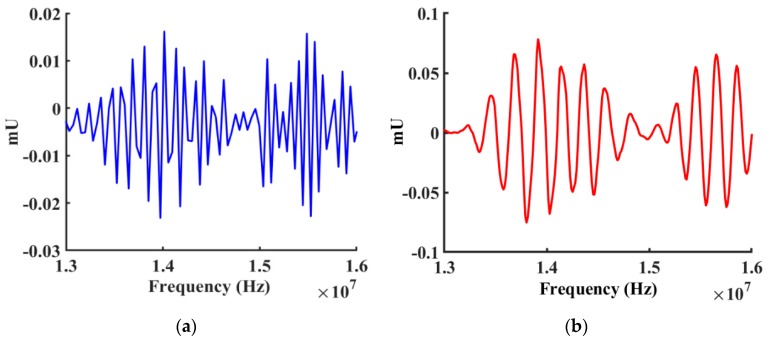
Comparison between simulated (Blue) and measured (Red) real magnitude responses for the device (with Cr IDTs only) from 12 MHz to 16 MHz. (**a**) Simulated real magnitude of the response from the design. (**b**) Measured real magnitude of the response from the fabricated device.

The imaginary part of z was converted to the imaginary magnitude of the response and compared with experimental results and plotted in Figure 11. The simulation results showed very similar characteristics for the first two peaks. The real part of z was converted to the real magnitude of admittance response and compared to the experimental results as shown in Figure 12.

### 3.3. Effect of Layer Sensitivity

For SAW-based biosensors, the sensitivity is a more important parameter for evaluating the overall performance of an acoustic sensor. To determine the mass sensitivity of the design, the frequency shift, phase shift, and insertion loss changes per mass loading are the primary parameters to evaluate. It is a sophisticated system for predicting the design and obtain a similar real sensitivity on test cases with a range of different mass loading. In our case, the actual wave propagation problem on the piezoelectric substrate involved multiple anisotropic layers, a double piezoelectricity layer, and three-dimensional wave diffraction. It is also not possible for other methods to analyze all the properties of the wave mode such as phase, electrical perturbation, mechanical mass loading, and wave transmission. The finite element method provides a more suitable and affordable method for characterizing the design compared to the much more costly and labor-intensive experimental approaches.

#### 3.3.1. Frequency Shift Detection

The fundamental biosensing technique using surface acoustic waves measures changes in propagation velocity, resonant frequency, phase angle, or to a lesser degree amplitude of reflection or transmission signals. Variations in these parameters of the acoustic wave can be attributed to intrinsic factors such as material properties: density, elasticity, phase transformation, viscosity, conductivity, permittivity, as well as changes in carrier concentration and mobility [35]. In our previous research [8,30], we show that the sensor’s structure is a delay line two-port resonator device, configured as a gained controlled RF oscillator system. In this oscillation setup system, the frequency is determined by the transfer function of the transducers and amplifiers through a closed-loop feedback configuration. After the two oscillation condition requirements are satisfied, which include loop gain over 0 dB and loop phase equal to 0 degrees, any change (Δv) in the phase velocity give rise to a frequency shift Δf in the output oscillation frequency f, given by [41]:

(6) $$\frac{\Delta f}{f_{0}}=\frac{\Delta v}{v_{0}}=\frac{1}{v}\left(\frac{\partial v}{\partial m}\Delta m+\frac{\partial v}{\partial \sigma }\Delta \sigma +\frac{\partial v}{\partial c}\Delta c+\frac{\partial v}{\partial \varepsilon }\Delta \varepsilon +\frac{\partial v}{\partial T}\Delta T+\frac{\partial v}{\partial P}\Delta P+\frac{\partial v}{\partial \rho }\Delta \rho +\cdots \right)$$

The above Equation (6) assumes that any other external perturbations listed below are negligibly small; where Δ m is the change in mass load, Δσ the change in conductivity, Δc the change in mechanical constant, Δε the change in dielectric constant, ΔT the change in temperature, ΔP the change in pressure, and Δρ the change in density.

The sensitivity of an acoustic wave sensor, *S_r_* to any external perturbation y is defined as:

(7) $$S_{r}=\underset{\Delta y\to 0}{\lim}\frac{\Delta f}{f\Delta y}=\frac{df}{fdy}$$

For the sensitivity specified based on other parameters such as phase, SWR, insertion loss and linear magnitude can be defined as:

(8) $$\frac{df}{fdy}=\frac{dp}{pdy}=\frac{dS}{Sdy}=\frac{dI}{Idy}=\frac{dL}{Ldy}$$

where *p* is the phase of the device, *S* is the value of the SWR, *I* is the insertion loss, and *L* is the linear magnitude of the response.

#### 3.3.2. Phase Shift Detection

The operation of the phase shift technique has been described in previous research, and the magnitude of the phase shift is more sensitive than the frequency shift [28,42,43]. The SAW device is configured as a delay line and fed by a radio frequency excitation signal. The phase of the signals at the input and output of the line are compared to obtain their difference *ϕ*, which is the phase delay of the acoustic line [41]:

(9) $$\varphi =2\pi \frac{1}{\lambda }=2\pi \frac{lf}{v}$$

(10) $$\Delta \varphi =-2\pi \frac{lf}{v}\frac{\Delta v}{v}=-\varphi_{0}\frac{\Delta v}{v}$$

Here, *l* is the length of the line corresponding to the center-to-center distance of the IDTs in Equations (9) and (10), *λ* is the acoustic wavelength at the operating frequency *f*, and *v* is the acoustic phase velocity. Any change Δv in the velocity is detected as a change Δφ in the phase delay $\varphi_{0}$ of the wave. The expression shows how the output signal, which is proportional to Δφ, can be magnified by increasing phase delay compared to frequency shift. On the other hand, it is necessary to consider the 2π period ranges in the response of the phase detector, which limit the upper levels of the dynamic range of the device.

#### 3.3.3. Sensitivity Comparison

Acoustic propagation mass sensing within multi-thin-layer systems is essential for optimizing gravimetric sensors. The mass sensitivity will vary by different thickness of the ZnO and IrO_2_ layer. A design created in COMSOL supports a parameter sweep to determine the relationship between thickness and sensitivity. All mass loadings were set up as a protein layer of approximately 35.64 ng protein at a fixed area (8 mm × 55 mm) with a thickness of 0.06 µm on top of the device as shown in Figure 1a, with varied layers of ZnO and IrO_2_ in terms of their thicknesses.

**Figure 13:**
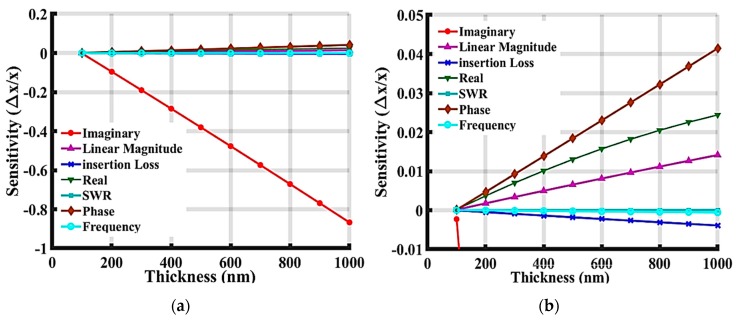
Comparison of normalized sensitivity defined based on different device parameters of the simulated SAW device with only ZnO layer. (**a**) Full view of the plot, and (**b**) zoomed figure without Imaginary data.

From Equation (8), the relative mass sensitivity can be measured in terms of different parameters such as phase, SWR, insertion loss, and linear magnitude. Figure 13 presents a comparison with respect to sensitivity based on the measurements of different parameters influencing sensitivity, including: the imaginary and real magnitude of the response, insertion loss S21, SWR, the total magnitude of phase, the linear magnitude of S21, and the frequency shift. After the protein layer was added to the top of the device surface, the received signal was compared to the one from the device without the added protein layer using Matlab^©^. The responses of the frequency shift, insertion loss change, and magnitude of phase have all been well studied in previous research. However, there is no prior work focusing on the sensitivity of other parameters. The verified model system developed herein can be used to define the device’s mass sensitivity with specific mass loading which is useful to predict the device’s capabilities and improve the achievable sensitivity. In Figure 13a,b, the imaginary phase shift shows the largest sensitivity to the mass loading. Even with only 100 nm ZnO layer on top of the device, it still has −0.0023 ppm of normalized sensitivity to mass loading compared to its frequency sensitivity which was only 1.57 × 10^−6^. The frequency in the oscillator system needs to adjust the loop phase to 0 degrees and then determine the frequency shift by comparing the two frequencies. Therefore, the data were imported to Matlab^©^ to match the loop phase, and were then compared to obtain the frequency shift. The relationship between the sensitivity of all these parameters indicated that the normalized sensitivity gradually decreased when it was defined based upon the imaginary magnitude of the response, magnitude of the phase, real magnitude of the response, linear magnitude S21, insertion loss S21 (dB), frequency with phase matched, or SWR. The magnitude of phase sensitivity was ~100 folds larger than the frequency shift at the ZnO thickness of 1000 µm. As the thickness of the guiding layer increased, the phase sensitivity increased compared to the frequency sensitivity, which can be confirmed by Equation (11).

(11) $$\nabla f=\frac{1}{\tau }\frac{\Delta ø}{360{}^{\circ}}=\frac{1}{\frac{L_{D}}{v_{D}}+\frac{L_{IDT}}{v_{IDT}}}\frac{\Delta ø}{360{}^{\circ}}$$

where *τ* is the delay time across the device, *v_D_* is the acoustic velocity in the delay line path, *v_IDT_* is the acoustic velocity in the IDT region, *L_D_* is the delay line path length, *L_IDT_* is the propagation path length in the IDT region, and ∆ø is the phase shift across the device. The velocity decrease caused by the guiding layer will increase the delay time. The simulation results confirmed that with the same amount of frequency shift at the device, phase shift should increase.

**Figure 14:**
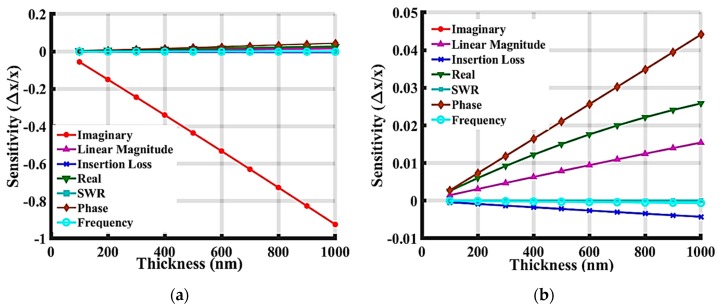
The simulated results based on different sensitivity parameters for SAW devices with 50 nm IrO_2_ layer and a ZnO layer of varied thicknesses. (**a**) Full view of the plot, and (**b**) zoomed figure without Imaginary data.

In Figure 14, the sensitivity of the response to the protein layer is compared, showing that all the sensitivities increased slightly compared to the results shown in Figure 13, where the 50 nm IrO_2_ layer was absent. This sensitivity increase has also been proven in our recent publication [35] by perturbation analysis, showing that a device with multiple guiding layers on top can achieve improved sensitivity. Figure 13 and Figure 14 show the phase sensitivity, the real part of admittance sensitivity, and the linear magnitude of S21. The device exhibits a passive sensitivity, meaning that as the mass loading is applied to the device, the values of these parameters increase. Negative sensitivity means that as the mass loading increases or guiding layer increases, the resonant phase, resonant insertion loss, and operating frequency of the device decrease.

**Figure 15:**
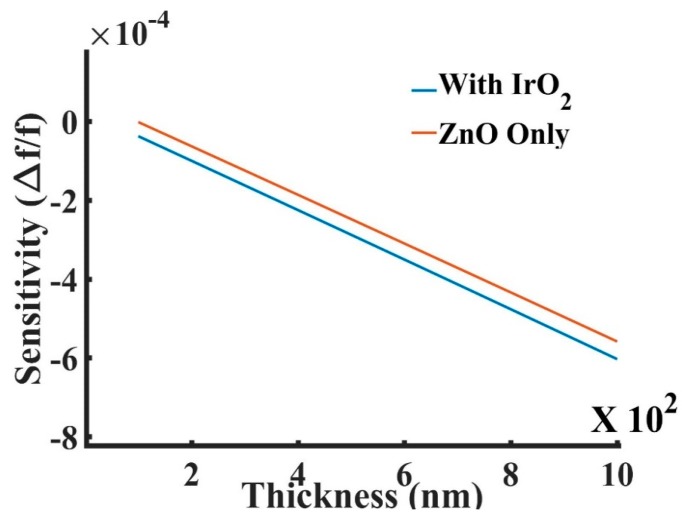
The frequency sensitivity comparison between a SAW-based device with only varying ZnO layer and a device with 50 nm IrO_2_ layer on top of the varying ZnO layer.

**Figure 16:**
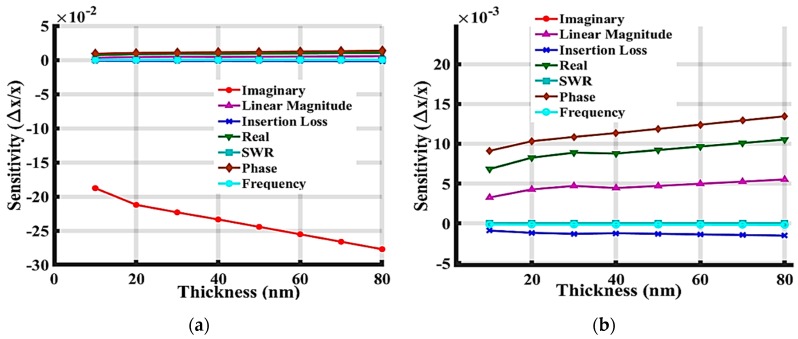
Simulation results of the SAW device with a 300 nm ZnO layer and an extra coating IrO_2_ layer of varied thickness from 10nm to 80nm. (**a**) Full view of the plot, and (**b**) zoomed figure without Imaginary data.

Figure 15 shows the frequency sensitivity comparison between the device with only a ZnO layer and the device with a multilayer of ZnO and a 50 nm-thick IrO_2_. The frequency sensitivity of the multilayer device was larger than that with only ZnO due to the additional IrO_2_ layer added on top, which increases the confinement of acoustic energy within the guiding ZnO layer. Figure 16 shows that the sensitivity increased as the thickness of the IrO_2_ increased from 10 nm to 80 nm.

## 4. Summary and Conclusions

In this study, we developed and evaluated a novel approach for characterizing and analyzing the SAW-based resonator device. This model was then verified by comparison to experimental data taken from fabricated devices. Our results show that the finite element model used herein reduces the computation requirements and time consumption while maintaining sufficient accuracy for the targeted research applications of acoustic sensor optimization to detect mass-loading effects. The simulation results of the sensor’s frequency spectrum were plotted, showing a clear trend and fit of the experimentally measured results. A thorough sensitivity analysis of the 3D model including S-parameters, reflection parameter, transmission parameter, and velocity phase were compared in this study. The results of this study can be used to determine and optimize the measurement configuration of SAW sensors. By comparing the sensitivity between the frequency, phase, and imaginary response of different guiding layers and structure designs, one can set up the appropriate measurement system such as an oscillatory circuit system or voltmeter and network analyzer system. However, a further study to reduce the spurious modes and increase the accuracy of the model will be investigated in the future. This study and its results will be used as feedback for the experimental device design in order to optimize the device in the future as this study shows that the simulation can accurately replicate experimental data.

## Figures and Tables

**Table 1:** Device IDT parameters used in both device fabrication and modeling.

PARAMETERS	SETTINGS
Wavelength (λ)	298 μm
Number of fingers	20 pairs
Finger width	74.5 μm
Wavelength of reflecting fingers	298 μm
Number of reflecting fingers	30 pairs
SAW velocity	4160 m/s

**Table 2:** Material properties used in COMSOL and Matlab^©^.

Material Properties	Units	Lithium Tantalate	ZnO	Cr	IrO_2_	Protein Fiber Layer
Density	(kg/m^3^)	4700	5680	7150	11660	1350
Young’s Modulus	(GPa)			279	322.8	0.07 [36]
Poisson’s ratio				0.21	0.33	0.44
Elastic stiffness $c_{11}^{E}$	×10^10^ (N/m^2^)	23.29	15.7			
Elastic stiffness $c_{12}^{E}$	×10^10^ (N/m^2^)	4.69	8.9			
Elastic stiffness $c_{13}^{E}$	×10^10^ (N/m^2^)	8.02	8.3			
Elastic stiffness $c_{13}^{E}$	×10^10^ (N/m^2^)	−1.1	0			
Elastic stiffness $c_{33}^{E}$	×10^10^ (N/m^2^)	27.53	20.8			
Elastic stiffness $c_{13}^{E}$	×10^10^ (N/m^2^)	8.02	4.3			
Elastic stiffness $c_{13}^{E}$	×10^10^ (N/m^2^)	9.30	4.42			
Piezoelectric coefficient *e*^15^	(C/m^2^)	2.596	−0.48			
Piezoelectric coefficient *e*^22^	(C/m^2^)	1.59	0			
Piezoelectric coefficient *e*^31^	(C/m^2^)	0.082	−0.57			
Piezoelectric coefficient *e*^33^	(C/m^2^)	1.882	1.32			

**Table 3:** The formula used for extracting values in the different Cartesian diagrams from the complex measurement.

Trace Format	Description	Formula
Lin Mag	Magnitude of z, unconverted	|z| = sqrt (x^2^ + y^2^)
Insertion Loss	Converted from z to S parameter	IL = −20 × log|S21| dB
Phase	Phase of z	φ (z) = arctan (y/x)
Real	Real part of z	Re(z) = x
Imag	Imaginary part of z	Im(z) = y
SWR	(Voltage) Standing Wave Ratio	SWR = (1 + |z|)/(1 − |z|)
